# Biomechanical analysis of barefoot walking and three different sports footwear in children aged between 4 and 6 years old

**DOI:** 10.1371/journal.pone.0291056

**Published:** 2023-09-05

**Authors:** Noelia Rodríguez López, Rafael Martínez Gómez, Mar Mínguez Valderrama, Adela García González, Julio C. de la Torre-Montero, Álvaro Pérez-Somarriba Moreno, Alberto J. Fidalgo-Herrera, Ana S. F. Ribeiro, Carlos López-Moreno, María Jesús Martínez-Beltrán

**Affiliations:** 1 Physical Therapy Unit. Puerta de Hierro University Hospital. Majadahonda, Madrid, Spain; 2 Faculty of Sport Sciences, Universidad Europea de Madrid, Madrid, Spain; 3 Center of childish development and early attention of Mancomunidad Sagra Alta, Toledo, Spain; 4 Comillas Pontifical University, Department of Health Sciences, San Juan de Dios School of Nursing and Physical Therapy, Madrid, Spain; 5 San Juan de Dios Foundation, Madrid, Spain; 6 Clinical Gait Analysis Department. Niño Jesús Pediatric University Hospital, Madrid, Spain; 7 Physical Therapy Department, Universidad Alfonso X, Villanueva de la Cañada, Madrid, Spain; Ningbo University, CHINA

## Abstract

The technological transformation and advertising utilized in the footwear industry significantly impact purchasing decisions. The gait properties, barefoot and with shoes, change depending on the footwear structure. The aim of this work is the biomechanical analysis of walking barefoot and with different sports shoes in a controlled group of 12 children between 4 and 6 years old. Kinematic and spatiotemporal variables were analyzed using a BTS motion capture analysis system with the Helen Hayes protocol. Previously, a survey was carried out with 262 families with children between 4 and 6 years old to justify the choice of footwear for this study. No significant differences were found between any of the measured conditions. The kinematic results showed significant differences in the ankle (right sagittal plane p = 0.04, left p < 0.01; right frontal plane p < 0.01, left p < 0.01), knee (right and left sagittal plane p < 0.01) and hip (right sagittal plane p < 0.01, left p = 0.04; right frontal plane p = 0.03). Additionally, the post hoc analysis revealed significant differences between barefoot gait and different footwear. The footwear used for this study and each one’s various characteristics are not preponderant in the spatiotemporal and kinematic parameters of the children’s gait. Thus, the footwear purchase may be conditioned by its design or composition and other properties may not be relevant.

## Introduction

Shoe manufacturers and health professionals advise that a shoe should not affect physiological foot function and motor development in young children, resembling barefoot walking [1, 2].

In children up to the age of five, parents make decisions in purchasing footwear, prioritizing product quality and ergonomics. Once children begin to intervene in the purchasing process, children’s brand licenses dominate the choices, giving way to sports brands [3]. Another factor to consider is the type of footwear manufactured. For children, the usual procedure is linear scaling, where different sizes are created from adult foot lasts, causing problems in the fit. These problems are generally related to width [4] because the child’s foot proportions do not correspond to those of the adult foot [5]. Therefore, a good fit of the last is necessary because it can influence the physiological development of the foot, even causing deformities in adulthood [6–9]. In addition, studies suggest that the shoe’s shape should resemble the foot’s shape and be designed according to the users’ measurements [10, 11].

Footwear also influences foot morphology and plantar arch [12, 13]. Individuals who have worn no shoes for extended periods appear to have broader feet, fewer toe and foot abnormalities, a higher foot arch, and lower hallux angles [14, 15]. In addition, the flexibility of habitually shod feet is known to be reduced [13–17]. Also, the prevalence of flatfoot was higher in those who started wearing shoes before the age of 6 years. Therefore, wearing shoes before that age may adversely affect the developing foot [8]. Also, the weight of shoes increases muscle activity in the medial gastrocnemius. Consequently, the shoe’s weight may be a critical factor when selecting shoes [12]. Another study concluded that the shoe model with the stiffer sole reduces plantar loading and decreases proprioception, consequently impacting gait development in its early acquisition stage [13].

Scientific evidence demonstrates that the gait acquisition process occurs in two phases and lasts several years. It was found that the postural capacity, which is necessary to control balance with the lower limb musculature, is achieved at 5–6 years old. Moreover, the gait is readjusted until it is stabilized at 7–8 years old when the control of the center of gravity displacement and the adult muscular activation pattern is complete. However, head control, integration of body parameters, and anticipatory postural adjustments require at least 8 years of gait experience to develop [12]. Additionally, locomotion activity experience determines lower limb movement control and balance until at least 10 years old. In this long and progressive process, antigravitational postural control, balance and movement coordination require considerable integration of sensorimotor components and motivational intention to drive movement, with higher functions governing that response [12, 16].

There are no procedures for choosing footwear compared to barefoot participants, which is a gap in children’s gait studies [1, 18]. It is unknown if the paucity of studies is due to the challenges gait analysis in this age group presents [16]. Interestingly, none of the footwear selection methods reported in some systematic reviews [1, 18] compared the outcomes with barefoot subjects.

Based on these findings, the present study was designed to evaluate the possible biomechanical differences between walking barefoot and with three different sports shoes in children aged 4–6 years.

## Methods

### Experimental design

This is an analytical, quantitative and quasi-experimental study. Each subject was measured from the biomechanical perspective of barefoot walking and with three types of footwear. Therefore, four tests were performed in random order on each subject 1:1.

### Participants

Participants were selected through probability sampling by convenience in educational institutions in collaboration with parents or legal guardians. Twelve subjects voluntarily participated (nine girls and three boys) in this pilot study (average data from the participants: age = 4.91 ± 0.66 years old; weight = 19.23 ± 2.34 kg; height = 109.70 ± 3.67 cm; BMI = 15.99 ± 1.90 kg/m^2^). The following inclusion criteria were applied: school children between 4 and 6 years old, with autonomous gait without limitations and absence of alterations in the range of mobility, strength, tone, or reflexes of the lower limbs (i.e., autonomous gait within the range of mobility). The subjects were assessed utilizing goniometry and functional tests [Silfverskiöld’s test (plantar flexors); Duncan-Ely’s test (rectus femoris); Thomas’s test (iliac psoas); Phelps’s test (adductors); Ober’s test (abductors); Too Many Toes sign, Jacks’s test, and Rodriguez Fonseca’s test (feet)] to confirm that they met these criteria. Subjects with a confirmed diagnosis of any neurological, traumatological, orthopedic, or rheumatological pathology were excluded. Those subjects with a body mass index out of normality were also excluded.

### Ethical considerations

This study followed the ethical recommendations of the Declaration of Helsinki and the Tokyo Declaration of the World Medical Association on medical research involving human subjects. The researchers adhered to the current Good Clinical Practice guidelines of the International Conference harmonization and the CRUE statement on good research practice at the university.

The research project was authorized by the accredited Clinical Research Ethics Committee of Hospital Clínico San Carlos (Madrid) (C.I. 19/229-E).

The parents were notified about the study through the participant information sheet and signed the informed consent form authorizing the child’s participation. The children received a similar adapted document. One parent always accompanied the child during the tests. The recordings made of the study subjects were stored together with all the study’s relevant data respecting the European and national laws, namely the European Regulation 2016/679 and the Ley Orgánica 3/2018, on Personal Data Protection and guarantee of digital rights and Biomedical Research Law 14/2007 and its 2016 update. A safety and hygiene protocol was created following the health recommendations due to the COVID-19 pandemic.

### Procedures

A prior online survey was conducted (between September 2019 and January 2020) with 262 families in the Community of Madrid with children between 4 and 6 years old to justify the choice of footwear for the study (S1 File). The results obtained from survey question number six responses showed that the most frequently used footwear during the school period was sports footwear (77.5%). Thus, three models of sports shoes were selected. The first model (Model 1) was selected based on the brand indicated by the families as the best footwear for their children (results obtained from the responses to survey question 9), with 36% of the responses. The second (Model 2) was chosen based on economic criteria within the lower range of the survey (results obtained from the responses to survey question 13). The research team picked the third model (Model 3), considering the characteristics of the materials and biomechanical criteria that would not alter the barefoot gait. More details are provided in the Results section.

The characteristics of the different footwear employed according to the protocol data for the evaluation of comfort in the footwear of the INESCOP footwear technology center are presented in Table 1.

**Table 1:** Analysis of the composition and functionality of the different shoes used in the study (INESCOP).

					Model 1	Model 2	Model 3	
CHARACTERISTICS				Units	Results	Results	Results	Recommended value
Shoe stiffness (dynamometer method)				N/45°	6.3	20.5	11.4	≤30^(1)^
Sweat management	Textile upper + lining	Permeability		mg cm^-2^.h	1.5	2.1	4.9	>2.0^(2)^
		Vapor coefficient		mg cm^-2^	15.1	17.3	39.7	>20^(2)^
	Insole + template	Water absorption		mg cm^-2^	134	13	212	>70^(2)^
		Water removal (24 h)		%	100	103	77	>80^(2)^
Electrical resistance (3 days/ 23 °C/ 50% RH) (1 kg electrode)				MΩ	10,449	118,256	18,665	≤5,000^(3)^
Energy management	Energy absorption			J	4.0	10.5	3.0	≥15^(4)^
	Impact absorption	Heel	Deceleration	m s^-2^	410	375	515	≤200^(5)^
			Penetration	mm	2.5	4.0	2.2	---^(6)^
			Returned energy	%	16	30	32	---^(6)^
		Thenar	Deceleration	m s^-2^	590	515	575	≤250^(5)^
			Penetration	mm	1.5	3.4	2.0	---^(6)^
			Returned energy	%	18	33	32	---^(6)^
Slip resistance (Friction coefficient) (Fv: 250N)	Tile E2/water	Flat		---	0.48	0.46	0.43	---
		Heel		---	0.43	0.45	0.31	---
	Tile E2/detergent	Flat		---	0.30^(^*^)^	0.30^(^*^)^	0.30^(^*^)^	≥0.30^(7)^
		Heel		---	0.32^(^*^)^	0.31^(^*^)^	0.21^(^*^)^	≥0.28^(7)^
Martindale abrasion	Dry	Vamp lining		Cycles	25,600^(a)^	25,600^(a)^	> 25,600	25,600^(8)^
		Insole		Cycles	> 25,600			
	Humid	Vamp lining		Cycles	6,400^(b)^	> 6,400	> 6,400	6,400^(8)^
		Insole		Cycles	> 6,400			
Sole hardness (Manual snapshot)				Shore A	70	65	75	---
Sole density				g cm^-3^	1.23	1.06	1.20	---
R. Sole abrasion				mm^3^	165 (PVC)	219 (TR)	97 (TPU)	≤150 (PVC) ≤180 (TR) ≤150 (TPU)
Thickness ^(9)^		Heel		mm	9.0	20.0	11.0	---
		Thenar		mm	9.0	11.5	10.0	---
		Drop		mm	0	8.5	1.0	---
Insole thickness		Heel		mm	5.0^(10)^	5.5	4.0	---
		Thenar		mm	5.0^(10)^	3.5	4.0	---
Floor thickness ^(11)^		Heel ^(12)^		mm	9.0	20.0	14.0	---
		Thenar ^(12)^		mm	9.0	11.5	12.0	---
		Drop		mm	0	8.5	2.0	---
Weight ^(13)^				G	132.2	195.7	146.3	---

INESCOP’s recommendation for comfortable children’s shoes.

Deviations of less than 20% from the recommended values were not considered significant when assessing the comfort of the footwear, provided that the wear test results were favorable.

(1) INESCOP recommendation for flexible footwear; (2) INESCOP recommendation for comfortable footwear for daily use; (3) INESCOP recommendation for footwear for daily use with antistatic characteristics; (4) Requirements established for soles of women’s/men’s footwear for daily use, in the Technical Report UNE-CEN ISO/TR 20880 IN "Calzado. Requisitos para componentes de calzado. Suelas"; (5) INESCOP recommendation for footwear for daily use; (6) To evaluate this property, the results of the use tests were considered; (7) Requirement UNE-CEN ISO/TR 20880 IN: "Calzado. Requisitos para componentes de calzado. Suelas"; (8) INESCOP recommendation for footwear for comfortable daily use, number of cycles without breakage; (9) Thicknesses: model 1: size 29 right; model 2: size 29 right; model 3: size 30 left; (10) Non-removable padded and lined sole; therefore, it acts as an insole; (11) Sole plus insole; (12) Includes everything under the foot; Thicknesses: model 1: size 29 right; model 2: size 29 right; model 3: size 30 left; (13) Average weight of right and left shoe of size 29; (a) Thread breakage; (b) Fabric breakage; (*) Correction factor of -0.07 for flat and -0.03 for the heel is applied.

**Fig 1:**
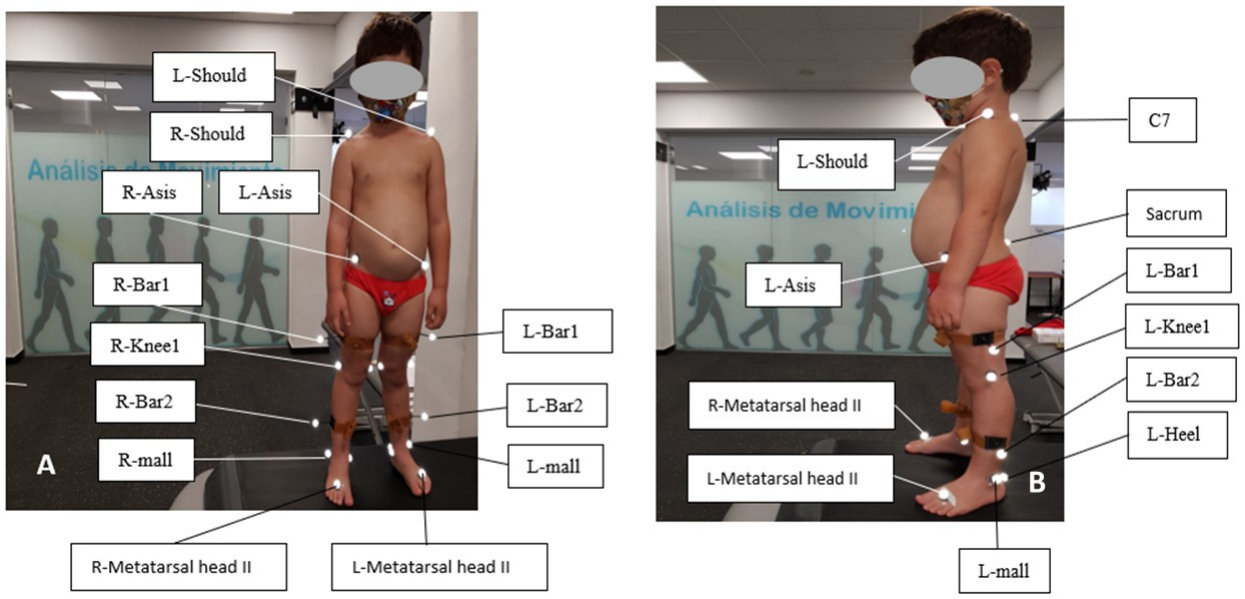
Markers placement according to the Helen Hayes MM protocol (A: Frontal view; B: Sagittal view).

The participants were conveniently summoned to take the measurements according to the following standardized protocol:

- Choice of shoe size: the length of the foot was measured, and 8% of this measurement was added, taking the shoe’s insole as a reference of the measurement [19].
- Anthropometric measurements: the child’s height, weight, and other parameters (e.g., width and depth of the pelvis, width of knees and ankles and leg length) were recorded for the movement analysis.
- Twenty-two markers were placed using the Helen Hayes MM protocol (Fig 1) [20].
- Gait analysis test: kinematics and spatiotemporal parameters were measured using a six-camera BTS motion analysis system (Smart-D 300 BTS Bioengineering, Italy) at a sampling rate of 240 Hz. An experienced motion analysis expert carried out these tests. For data collection, necessary trials were performed to obtain both limbs’ kinematic and spatiotemporal values. Four walks were conducted to obtain a representative average gait. This analysis was replicated for the barefoot gait and the three footwear models, tracking, processing, and visualizing the data using the BTS SMART Clinic software (BTS Bioengineering, Italy).

The procedure took approximately 120 minutes for each subject. Adequate rest was provided between measurements.

### Variables studied

The variables collected and analyzed by the motion analysis system included: spatiotemporal (13 variables) and kinematic parameters (40 variables) (Table 2).

**Table 2:** Variables analyzed by the motion analysis system.

SPATIOTEMPORAL parameters (stance phase (%); swing phase (%); double support (%); Velocity%height (m s^-1^); stride length%height; swing support phase (m); gait deviation index (numeric scale))		KINEMATIC parameters (°)	
**R stance phase**	sRSTANCE	R Ankle dorsi-plantar flex	aRAFE
**R swing phase**	sRSWING	L Ankle dorsi-plantar flex	aLAFE
**R double support**	sRDBLSTANCE	R Ft progression angle	aRAIE
**L stance phase**	sLSTANCE	L Ft progression angle	aLAIE
**L swing phase**	sLSWING	R Knee flex-extension	aRKFE
**L double support**	sLDBLSTANCE	R Knee valgus-varus	aRKAA
**Velocity%height**	VelMEAN%HEIGHT/S	R Knee rotation	aRKIE
**L stride length%height**	LSTRIDE%HEIGHT	L Knee flex-extension	aLKFE
**R stride length%height**	RSTRIDE%HEIGHT	L Knee valgus-varus	aLKAA
**R swing support phase**	sRSWINGSTANCE	L Knee rotation	aLKIE
**L swing support phase**	sLSWINGSTANCE	R Hip flex-ext	aRHPFE
**R gait deviation index**	R GDI	R Hip ab-adduction	aRHPAA
**L gait deviation index**	L GDI	R Hip rotation	aRHPIE
		L Hip flex-ext	aLHPFE
		L Hip ab-adduction	aLHPAA
		L Hip rotation	aLHPIE
		R pelvic tilt	aRPTILT
		R pelvic obliquity	aRPOBLI
		R pelvic Rotation	aRPROT
		L pelvic tilt	aLPTILT
		L pelvic obliquity	aLPOBLI
		L pelvic Rotation	aLPROT
		R Trunk rotation	aRSHROT
		L Trunk Rotation	aLSHROT
		R spine lateral Bending	aRSPML
		R spine rotation	aRSPIE
		L spine lateral Bending	aLSPML
		L spine rotation	aLSPIE
		R trunk tilt	aRSHTILT
		R trunk obliquity	aRSHOBLI
		L trunk tilt	aLSHTILT
		L trunk obliquity	aLSHOBLI
		R trunk tilt relative	aRSHTILTOFF
		L trunk tilt relative	aLSHTILTOFF
		R trunk obliquity relative	aRSHOBLIOFF
		L trunk obliquity relative	aLSHOBLIOFF
		R trunk rotation relative	aRSHROTOFF
		L trunk rotation relative	aLSHROTOFF
		R spine flex-extension	aRSPFE
		L spine flex-extension	aLSPFE

### Statistical analysis

The Shapiro Wilk test was performed to check the normality of the variables using the non-parametric statistical mapping technique (SnPM) with MATLAB^®^ version 2020a software (The MathWorks Inc., Natick, Massachusetts).

The MANOVA test was performed using the SPSS 26.0 software (IBM Corp., Armonk, NY) to test the spatiotemporal parameters. For the remaining variables, repeated-measures ANOVA was used for data with a normal distribution. The Kruskal Wallis test was used for the variables that did not display a normal distribution using the Statistical non-Parametric Mapping (SnPM) package.

All continuous variables were tested for normal distribution. When this distribution was not followed the non-parametric alternatives for SPM analysis were used. The SPM/SnPM statistical package [21, 22] was imported into MATLAB^®^, which was validated for biomechanical data analysis. When performed, post hoc tests (repeated measures t or Friedman’s test) were conducted with a Bonferroni correction of 0.05/4 = 0.0125. For all tests, an alpha error of 0.05 was assumed.

## Results

From the survey to justify the choice of footwear, it was found that sports footwear is the most used for the age group studied. Additionally, it was observed that the primary aspects families consider when choosing footwear include the manufacturing materials (leather, breathable fabric), the sole type (soft, hard, non-slip), price, and insole shape. The shoe aesthetics were a secondary factor. Families also trust the shoe brand to determine if a shoe is healthy. They also take the salesperson’s advice, and few rely on scientific information.

Concerning the motion analysis variables of the study, no differences were detected for the spatiotemporal parameters among any of the conditions tested [MANOVA results were F (36, 98.23) = 1.30, p = 0.15; Wilks’ Λ = 0.31] (Table 3).

**Table 3:** Spatiotemporal parameters (N = 12).

	Barefoot Mean ± SD	Model 1 Mean ± SD	Model 2 Mean ± SD	Model 3 Mean ± SD	Value	F	Hypothesis DF	Error DF	p-value (Wilks’ Lambda)
**sRSTANCE (%)**	58.30 ± 1.36	59.52 ± 1.66	60.39 ± 1.17	60.37 ± 1.99	0.314	1.308	36	98.23	0.151
**Srswing (%)**	41.67 ± 1.36	40.48 ± 1.66	39.61 ± 1.17	39.63 ± 1.99					
**Srdblstance (%)**	8.65 ± 1.39	9.28 ± 1.61	10.54 ± 1.68	10.27 ± 1.84					
**Slstance (%)**	58.95 ± 1.42	59.39 ±1.81	60.62 ±1.38	60.23 ± 1.92					
**Slswing (%)**	41.05 ± 1.42	40.58 ± 1.78	39.38 ± 1.38	39.80 ± 1.93					
**sLDBLSTANCE**	8.66 ± 1.33	9.54 ± 1.99	10.47 ±1.43	10.33 ± 2.36					
**VelMEAN%HEIGHT/S (m s** ^**-1**^ **)**	103.84 ± 8.31	106.55 ± 11.20	107.84 ± 8.48	105.12 ± 14.30					
**LSTRIDE%HEIGHT (m)**	82.71 ± 5.55	92.59 ± 8.05	95.26 ± 7.77	90.57 ± 7.30					
**RSTRIDE%HEIGHT (m)**	82.49 ± 5.20	92.41 ± 8.56	95.69 ± 7.98	90.49 ± 7.40					
**Srswingstance (m)**	41.11 ± 1.53	40.85 ± 1.55	39.30 ± 1.30	39.75 ± 2.23					
**Slswingstance (m)**	41.63 ± 1.24	40.24 ± 2.34	39.72 ± 1.61	39.72 ± 1.89					
**R GDI**	96.66 ± 9.41	92.43 ± 7.27	91.70 ± 6.78	92.56 ± 6.49					
**L GDI**	96.75 ± 6.59	93.86 ± 7.11	93.99 ± 6.11	93.65 ± 6.05					

**Fig 2:**
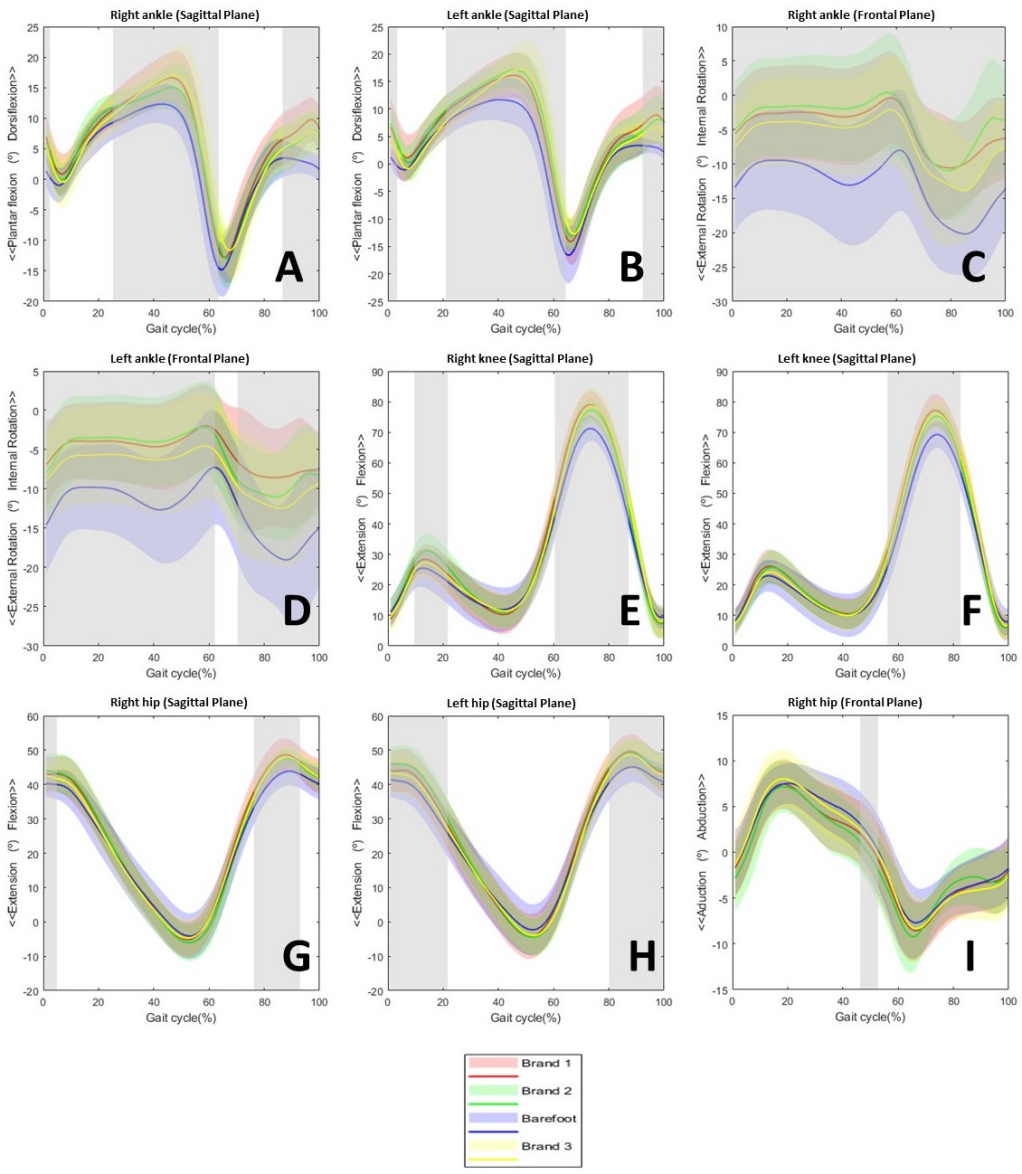
Statistically significant results for kinematic variables (N = 12).

Abbreviation definitions in Table 2,

The kinematics of the ankle, knee, hip, pelvis, and trunk were investigated as a component of this study. As shown in Fig 2, considerable contrasts were only identified for the ankle, knee, and hip.

For the right ankle in the sagittal plane, the critical threshold of 5.55 was crossed by three supra-threshold clusters with probability values of p = 0.04, < 0.01, < 0.01 between 0–2.39%, 25.20–63.44%, 86.64–100% of the gait cycle (Fig 2A). Post hoc examination revealed that those distinctions must be made between each model and the shoeless condition. Moreover, for the left ankle in the sagittal plane, the critical threshold 5.20 was crossed by three supra-threshold clusters with probability values of p < 0.01, < 0.01, < 0.01 between 0–3.39%, 21.01–64.35%, 92.24–100% of the gait cycle (Fig 2B). Post hoc analysis only revealed differences between models 1 and 2 compared to the barefoot condition and model 2 compared to model 3.

Examination of the right ankle in the frontal plane showed that the critical threshold 4.77 was traversed by a supra-threshold cluster with probability values of p < 0.01 between 0% and 100% of the gait cycle (Fig 2C). Post hoc examination identified distinctions between the shoeless condition and each model but not between the shoe models.

In the frontal plane of the left ankle, the critical threshold 4.86 was intersected by two supra-threshold clusters with probability values of p < 0.01, < 0.01 between 0–62.13%, and 70.39–100% of the gait cycle (Fig 2D). Again, the differences are only detected between the barefoot condition and each model, but not between them.

In the sagittal plane of the right knee, the critical threshold 5.27 was crossed by two supra-threshold clusters with probability values of p < 0.01, < 0.01 between 9.59–21.67%, 60.43–87.13% of the gait cycle (Fig 2E). Post hoc analysis revealed differences between the barefoot condition and shoe models 1 and 2.

Analysis of the left knee in the sagittal plane found that the critical threshold 5.40 was traversed by a supra-threshold cluster with probability values of p < 0.01 between 56.15–82.57% of the gait cycle (Fig 2F). All differences found in the post hoc examination were between each shoe model and the shoeless condition.

In the right hip in the sagittal plane, the critical threshold 4.68 was crossed by two supra-threshold clusters with probability values of p < 0.01, < 0.01 between 0–21.62%, 80.08–100% of the gait cycle (Fig 2G). Post hoc investigation shows that differences were only pertinent between the shoeless condition and shoe models 1 and 2.

At the left hip in the upside view, the critical threshold 4.98 was passed by two supra-threshold clusters with probability values of p = 0.04, 0.01 between 0–4.91%, 76.23–92.95% of the gait cycle (Fig 2H). Post hoc examination showed differences when comparing models 2 and 3 with the barefoot conditions but not for the others.

In the right hip’s frontal plane, the threshold value of 5.04 was exceeded by a supra-threshold cluster with a p = 0.03 from 46.24–52.75% of the gait cycle (Fig 2I).

## Discussion

The literature indicates that the high replacement of children footwear makes quality and price the decisive purchasing factors [3]. Additionally, the prestige of the brand and the advice of retailers and friends influence the final purchase decision [3, 23]. Previous reports also point out that influences are generated through cinema and television in the 5–9 age group, and advice from salespeople is more critical in the under-5 age group [3].

Regarding the spatiotemporal parameters, our results revealed no significant differences between the studied conditions. However, studies report substantial changes in the support phases in speed, cadence, and stride length in gait. Nonetheless, these studies did not specify the technical characteristics of the shoes with which they were compared [1, 18].

In our study, the Gait Deviation Index (GDI), although not statistically significant, is lower in the gait with shoes compared to the barefoot gait, which is clinically relevant and may indicate a worse gait pattern [24, 25].

Concerning the kinematic parameters in the sagittal plane, significant differences between walking barefoot or wearing shoes were found, especially for the ankle, knee, and hip [1, 18]. It should be noted that we obtained variability in our results between both lower limbs (right-left) with the different footwear. Pulido-Valdeolivas et al. [26] also demonstrated variability in barefoot walking between both lower limbs in healthy schoolchildren.

Regarding the kinematics of the ankle joint, significant differences in all stance phases between barefoot walking and footwear with increased ankle dorsiflexion were detected, as demonstrated by Chen et al. [12]. This observation results from the ankle having greater dorsiflexion and becoming weight-bearing with shoe cushioning. Increased dorsiflexion augments deceleration by increasing the response to load and will be absorbed by the shoe’s cushioning. A similar result was found in adult runners when comparing barefoot and footwear running [27].

Although the results were not significant in the transversal plane, it was observed that internal progression increased with shoes compared to barefoot walking on both sides. Considering that none of the children had previous orthopedic alterations or internal progression in their feet, it is surprising that their internal progression increases with shoes. However, similar results were previously reported by other authors [28, 29].

In the knee, significant changes in peak flexion in oscillation occur between shoe models 1 and 2 and barefoot walking. This result is probably related to the increase in the ankles’ dorsiflexion in the swing phase as a stepping strategy to avoid the ground or the shoe’s weight generating a greater strength of the ankle dorsiflexors and hip flexors at a proprioceptive level [28, 29]. These increases in flexion correlate with the increase in knee flexion in response to loading, which is when body weight decelerates. In this sense, it may be related to the difference in heights between barefoot and shoe walking. The increase in ankle dorsiflexion in oscillation at the knee leads us to believe that the distance of the first toe from the ground in oscillation increases to avoid stumbling [30, 31]. In contrast, the kinematic reported by Chen et al. [12] revealed that foot type (flat or healthy) and shoe type affect all lower limb joints except the knee joint.

Regarding the hip joint, we observed a significant increase in flexion at initial contact in the left hip between barefoot and shoe models 1 and 2. However, in the right hip, we detected differences between shoe models 2 and 3. Compared to barefoot, the flexion peak in oscillation is also increased, which correlates with an enhanced knee flexion peak and dorsiflexion in the oscillation of the ankle. It is plausible that this is a strategy to increase the toe-floor distance with shoes [28–30]. Similar data were also reported by [32], who demonstrated increased hip flexion with shoe use in the adult population. On the other hand, a study comparing shod and barefoot walking in healthy children, albeit with flat feet, observed attenuation in hip flexion in both groups [12].

There is substantial heterogeneity in the studies comparing barefoot walking and footwear biomechanics in the pediatric population. Due to the type of footwear, materials, gait speeds and age group, it is concluded that additional studies should be conducted with more population groups and footwear types with larger sample sizes, to assess the impact of these variables on gait [1, 18, 32].

## Study limitations

The variables analyzed showed no sensitivity to changes in footwear. Although this information is relevant, we cannot affirm that no changes have occurred at the level of load distribution in the foot or the intrinsic kinematics of the foot. The study was conducted on a controlled and adherent surface, so we do not know if the performance of the shoes could differ in other situations such as with reduced adherence or unstable terrain. Moreover, the long-term performance of these shoes has not been assessed, and shoe deterioration could influence performance over time. On the other hand, the comfort of these shoes is also an important variable to analyze. Given all these limitations, we believe that follow-up studies could be conducted to explore all these new perspectives. Another possible limitation of this study was the marker positioning on the barefoot vs. shod foot.

## Conclusion

In summary, we found modifications between barefoot walking and three different footwear that influence the kinematic parameters of the ankle, knee, and hip; however, significant differences were not observed. It is anticipated that these results could impact the purchasing processes of children’s footwear. The selection of footwear and its different characteristics may not be preponderant in the kinematic parameters of the children’ gait. Nevertheless, further studies are needed to know how different types of footwear influence the pediatric population. There are also multiple orthopedic pathologies related to movement disorders, such as torsional deformities, tiptoe gait, and valgus flatfoot, which occur from a very early age and whose origin is unknown. Literature explaining the causes or what factors may be related to these pathologies is scarce. The biomechanical impact of shoes on pediatric gait has yet to be described, and more studies are needed.

## Supporting information

S1 File Survey used in the study. (DOCX)
