# Supplementary material for: Biomechanical analysis of barefoot walking and three different sports footwear in children aged between 4 and 6 years old
Source: PLoS One. 2023 Sep 5;18(9):e0291056. doi: 10.1371/journal.pone.0291056 (PMC10479898; doi:10.1371/journal.pone.0291056)
Supplement: S1 File — (DOCX) [file pone.0291056.s001.docx]

**Supporting Information**

**S1.** **Survey used in the study.**

1. Parents' educational level
   1. No studies
   2. Primary education
   3. Secondary education (Baccalaureate/Vocational training)
   4. Higher education (University)
2. What is the household's annual income?
   1. < 10,000 €
   2. 10,001-20,000 €
   3. 20,001-30,000 €
   4. 30,001-40,000 €
   5. 40,001-50,000 €
   6. 50,001-60,000 €
   7. > 60,001 €
   8. Does not know/does not answer
3. Child's age
   1. 4 years old
   2. 5 years old
   3. 6 years old
4. Gender
   1. Boy
   2. Girl
5. Child's shoe size
   1. 25
   2. 26
   3. 27
   4. 28
   5. 29
   6. 30
   7. 31
   8. Other
6. What shoes does your child usually or most frequently wear during school??
   1. sports shoes
   2. School shoes
   3. Dress shoes (moccasins, Mary Janes, other)
   4. Ankle-high boots
   5. Mid-leg boots
   6. Ergonomic footwear
7. Rate from 1 to 5 the importance you give to different aspects of the child's shoes when buying them (1 not very important and 5 very important)

| Aesthetics of the shoe (colors, external design, other) | 1 | 2 | 3 | 4 | 5 |
| --- | --- | --- | --- | --- | --- |
| Materials of construction (leather, fabric, breathable, other) | 1 | 2 | 3 | 4 | 5 |
| Price | 1 | 2 | 3 | 4 | 5 |
| A well-known brand | 1 | 2 | 3 | 4 | 5 |
| Type of sole (soft, hard, non-slip, other) | 1 | 2 | 3 | 4 | 5 |
| Shape of the insole (flat, with arch support, other) | 1 | 2 | 3 | 4 | 5 |

1. Which of these children's footwear brands do you consider supported by scientific studies, scientific societies and/or universities?
   1. Biomecanics
   2. Adidas
   3. Nike
   4. Pablosky
   5. Garvalin
   6. Reebok
   7. Chicco
   8. Do not know of any
2. What do you think is the best footwear brand for your child's feet?
   1. Biomecanics
   2. Adidas
   3. Nike
   4. Pablosky
   5. Garvalin
   6. Reebok
   7. Chicco
   8. Other
3. On what basis do you know if a shoe is healthy for your child? Please check the most important one.
   1. Guarantee of brand prestige
   2. Scientific disclosures
   3. Publicity
   4. Taking advice from family, friends, or others.
   5. Taking advice from the salesperson
4. In the process of choosing footwear, to what extent does your child's opinion play a role?

| A little | 1 | 2 | 3 | 4 | 5 | A lot |
| --- | --- | --- | --- | --- | --- | --- |

1. How healthy would you say the shoes you usually buy are for your child's feet?

| Not healthy | 1 | 2 | 3 | 4 | 5 | Very healthy |
| --- | --- | --- | --- | --- | --- | --- |

1. What price would you be willing to pay for healthy shoes for your child?
2. 0-24.99 €
3. 25-49.99€
4. 50-74.99€
5. > 75 €
